# Magnetic Nanosystems as a Therapeutic Tool to Combat Pathogenic Fungi

**DOI:** 10.34172/apb.2020.063

**Published:** 2020-08-09

**Authors:** Heba Salah Abbas, Akilandeswari Krishnan

**Affiliations:** ^1^National Organization for Drug Control and Research, Cairo, Egypt.; ^2^Scientist Under Scheme of Asian Research Training Fellowship for Developing Country (RTF-DCS), FICCI, NewDelhi, India.; ^3^Department of Pharmaceutical Technology, Bharathidasan Institute of Technology, Anna University, Tiruchirappalli-620024. Tamilnadu, India.

**Keywords:** Candida infections, Iron NPs synthesis, Magnetic nanosystems, Surface modification, Antibacterial, Antifungal mechanism

## Abstract

The overuse of antibiotics is the main reason for the expansion of multidrug-resistant microorganisms, especially, pathogenic fungi, such as Candida albicans and others. Nanotechnology provides an excellent therapeutic tool for pathogenic fungi. Several reports focused on metal oxide nanoparticles, especially, iron oxide nanoparticles due to their extensive applications such as targeted drug delivery. Using biological entities for iron oxide nanoparticle synthesis attracted many concerns for being eco-friendly, and inexpensive. The fusion of biologically active substances reduced and stabilized nanoparticles. Recently, the advancement and challenges for surface engineered magnetic nanoparticles are reviewed for improving their properties and compatibility. Other metals on the surface nanoparticles can enhance their biological and antimicrobial activities against pathogenic fungi. Furthermore, conjugation of antifungal drugs to magnetic nanoparticulate increases their antifungal effect, antibiofilm properties, and reduces their undesirable effects. In this review, we discuss different routes for the synthesis of iron oxide nanoparticles, surface coating manipulation, their applications as antimicrobials, and their mode of action.

## Introduction

Recently, the overload of fungal diseases causes 1 500 000 global deaths every year.^1^ *Candida* species produces severe infections that may involve damage of crucial organs.^2^ One hundred and fifty various species of the genus Candida were recognized including *C. albicans, C. krusei , C. glabrata, C. tropicalis, C. parapsilosis , C. lusitaniae , C. dubliniensis , C. Kefir, C. guilliermondii* and *C. stellatoidea .* They can cause human infectionsand the most invasive are infections caused by*C. albicans.*^3-5^ *C. albicans* is one of the normal floras which are found in vagina, mouth, and dorsum of the tongue. The increase of candidiasis occurrence is closely related to the immunodeficiency syndrome in human. *C. albicans* can cause systemic infections in immunocompromised patients, such as endocarditis, and lung and brain infections. Even any change in the commensal organisms of the intestine, because of antibiotic treatments, leads to intestinal candidiasis. Infants can also be infected by vaginal candidiasis during delivery and their contact with the vagina.^6,7^

In most populated countries such as Egypt, around 1 307 766 adult women suffered from vulvovaginal candidiasis in 2012. Also, candidaemia and intra-abdominal candidiasis were estimated by 4127 and 806 cases.^8^ In India, high incidence of candidemia was recorded in an intensive care unit.^9^ In China, *Candida auris* has been isolated from hospital women but, it was less virulent than *C. albicans.* The emergence of multidrug-resistant *C. auris* and its relation with high mortality is a critical issue. ^10^

The virulence factors of*Candida* species which are responsible for pathogenicity include their effect on the host defenses by adherence, biofilm creation or/and production of proteases, phospholipases, and others that damage the host tissue.^11^ Various antifungal drugs are available for the treatment of candidiasis such as amphotericin B but, it has poisonous effects. Fluconazole is safer but, certain *Candida* species are resistant to it.^12^ The emergence of resistance against pathogenic fungi to fluconazole and amphotericin B is a major public health concern. There is an urgent demand to develop new antifungal agents.

Nanotechnology draws the attention of many researchers due to its various applications. The activities of nanoparticles largely depend on particle size. The properties of nanoparticles can change by decreasing the particle size at nanometer scale.^13-15^ Green nanotechnology employs the use of biological sources such as microorganisms, plants or algae extract for the synthesis of nanomaterials. Green approaches produce safe and eco-friendly nanomaterials due to the absence of toxic substances during synthesis.^16^

Magnetic nanoparticles are oneof the most important metal oxides because of their widespread applications in biotechnology and medicine.^17,18^ Recently, the encapsulation of fungal drug in nanoparticle schemes offers an innovative alternative approach that promotes therapeutic efficiency and decreases the inappropriate side effects of the drugs. Limited studies were carried on the antifungal activities of biosynthesized Iron oxide nanoparticles. The antifungal activity of biosynthesized iron oxide nanoparticles was previously investigated.^19^ Iron oxide nanoparticles cause inhibition for growth and spore germination of *Trichothecium roseum , Cladosporium herbarum , Penicillium chrysogenum , Alternaria alternata* and *Aspergillus niger* . The continual resistance of microorganisms led to advancement of chitosan coated iron oxide nanoparticles as new antimicrobial agents against *Escherichia coli* , *Bacillus subtilis* , *C. albicans* , *A. niger* and, *Fusarium solani.*^20^ Our study aims to discuss routes for synthesis of iron oxide nanoparticles, surface coating manipulation and, their potential use as new antifungal agents.

## Methods for Synthesis of iron oxide nanoparticles

### Physical methods

**Figure 1:**
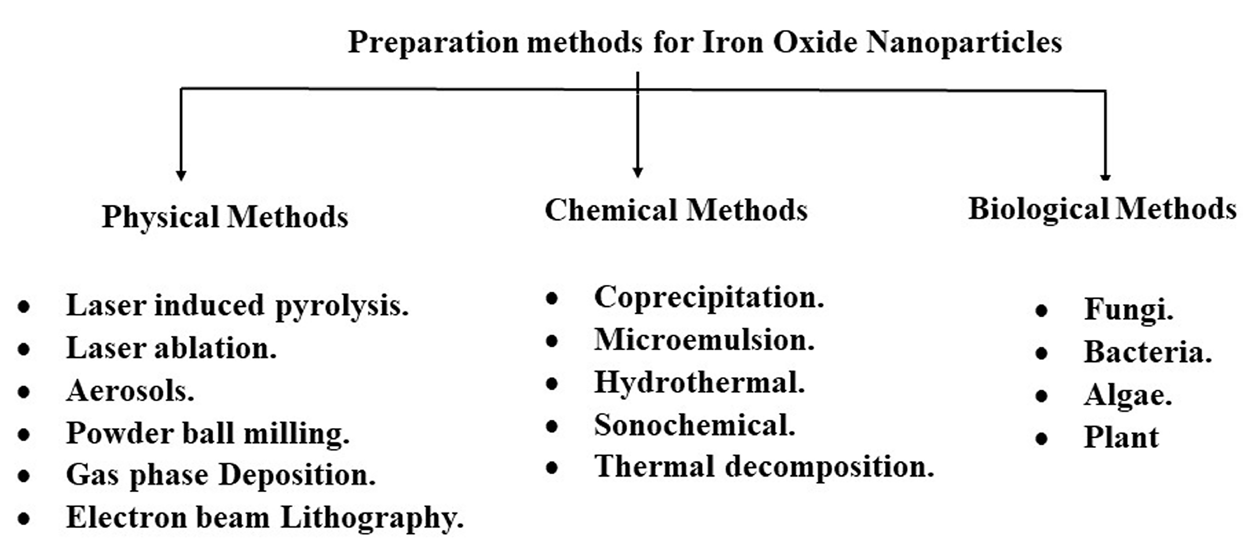
Flow chart for preparation methods of Iron oxide nanoparticles.

Ironoxide nanoparticlescan be synthesized via various techniques such as chemical, physical, and biological techniques (Figure 1). There are different methods for physical synthesis of Iron oxide nanoparticles such as pyrolysis, laser ablation, etc.

*Laser ablation method* depends on the solvent used whether it is organic, or inorganic solvent such as ethanol, or acetone. In general, ethanol and acetone are better than organic solvents because organic solvents can elaborate various by-products, with different physical and chemical characters, which show influence on nanoparticles stability.^21^ Using Polymers during the synthesis of iron oxide nanoparticles can control their size and distribution. This stabilized iron oxide nanoparticles showed good antimicrobial characterstics.^22^

In *spray pyrolysis or gas/aerosol method* , ferric salt solution and a reducing agent sprayed and the aerosol solute condensed during the solvent evaporation. The yield percentage is very low and the equipment for this method is very expensive.^23,24^ The most disadvantage of this method is the uncontrollable size of nanoparticle in nanometer range.^25^

Also, Kang and Rhee have studied the impact of pressure (60 torr) and 800°C temperature on ultrasonic spray pyrolysis by using acetate and nitrate solutions for the synthesis of manganese, nickel, and copper oxide. The products were hollow shaped submicron particles with large crystalline size (>40 nm) and nanoparticles with small crystalline size (<10 nm).^26^ In other study, Ozcelik and Ergun elucidated that the crystallinity of the spherical iron oxide increased by increasing temperature to 1100°C.^27^

### Chemical methods

Various techniques are documented for the chemical synthesis of nanoparticles such as coprecipitation, microemulsion, hydrothermal, thermal decomposition, and sonochemical methods. They are categorized by their simplicity, low-cost, and high yield of nanoparticles with controlled morphology.

In*coprecipitation* , iron oxide nanoparticles are synthesized by adding base into ferric chloride solution followed by precipitation black coloured magnetite. Magnetite precipitates in alkali conditions (pH 9-14) and in the absence of oxygen. Otherwise, it is oxidized into hydroxide form as in the subsequent equation: -

Fe_3_O_4_ + 0:25O_2_ + 4:5H_2_O → 3Fe (OH)^ 3+^

The bubbling of nitrogen gas during the process of synthesis protects iron oxide nanoparticles from oxidation and decreases their size. Also, the coating of nanoparticles by using organic and inorganic molecules prevents their agglomeration and oxidation.

The kind of salt precursor, ferrous/ferric ratio (1:2), pH, ionic strength, temperature, and the bubbling of nitrogen gas can influence the morphology of iron oxide nanoparticles.^23,28^

Also, Nazari et al used wool fabrics and butane tetracarboxylic as a stabilizer for iron oxide nanoparticles to get better results as antifungals against *C. albicans* .^29^

The*hydrothermal method* requires high pressure (>2000 psi) and temperature (>200°C). The reaction depends on hydrolyzing the metal salt by water in autoclave or reactor. However, this method takes long time and elevated temperature for synthesis and this causes effect on the size and morphology of metal oxide nanoparticulate.^25, 30-32^

In *microemulsion method (two phases method),* the nano-water droplet disperses in oil and is stabilized by surfactant. The surfactant type may be cationic, anionic, or none-anionic form. The core advantage of this method is the production of diverse nanoparticles by changing reaction conditions like introducing an oil phase or changing the quantity of surfactant.^33^ However, the disadvantages are: low temperature, large amount of oil that make large-scale production difficult, and the effect of residual surfactant on nanoparticles properties.^34-36^

In *thermal decomposition method* , iron salt precursors decompose thermally without oxygen and produce a high yield of Iron oxide nanoparticles. However, the product is mixture of nano-iron oxide phases with crystal defects, and also, its hydrophobic nature needs additional stages to be compatible with hydrophilic surface.^37,38^ During the thermal decomposition process, Unni et al synthesized a single nano-iron oxide phase with limited defect by addition of oxygen.^39^

*In the sonochemical method* , iron precursor such as ferric chloride hexahydrate is decomposed by high intensity of ultrasonication then polymers are added for capping and stopping nanoparticles growth. Cavitation can occur due to ultrasonic irradiation, with a consequent increase in temperature to reach 5000°C and of pressure to exceed 1800 kPa, causing anomalous chemical reactions (Table 1).^39,40^

**Table 1:** Physical and chemical preparation methods for iron oxide nanoparticles, types of particles, morphology, advantages, and disadvantages of different methods

**Methods**	**Nanoparticles Morphology**	**Types of** **Particles**	**Advantage**	**Disadvantage**	**References**
Physical-laser ablation method	Spherical, 20-100 nm	Maghemite- Hematite	Stable with a narrow size distribution only in Polymeric solution	Uncontrolled size in water solution	22
Physical-spray pyrolysis	Spherical, 70-675 nm	Hematite	Uniform morphology	Crystallinity increases by High temperature (1100^o^C)	27
Chemical-coprecipitation	Nanocubes (7.8 ± 0.05 nm) and nanorod (6.3 ± 0.2 nm)	Magnetite	Small sized nanoparticles, Simple reaction conditions		28
Chemical-hydrothermal	Spherical (15.6±4.0 nm) or Rhombic (27.4±7.0 nm)	Maghemite	Small sized nanoparticles	High pressure and temperature requirements. It easily affected by precursor concentration	32
Chemical-microemulson	Spherical, <10 nm	Magnetite or Maghemite	Diverse nanoparticles		33
Chemical-thermal decomposition	Spherical	Mixed phases	High yield	Poor and crystal defects. Hydrophobic nature.	39
Chemical-sonochemical	Spherical >19 nm	Hematite	Small size	High temperature and pressure	41

### Biological methods

**Figure 2:**
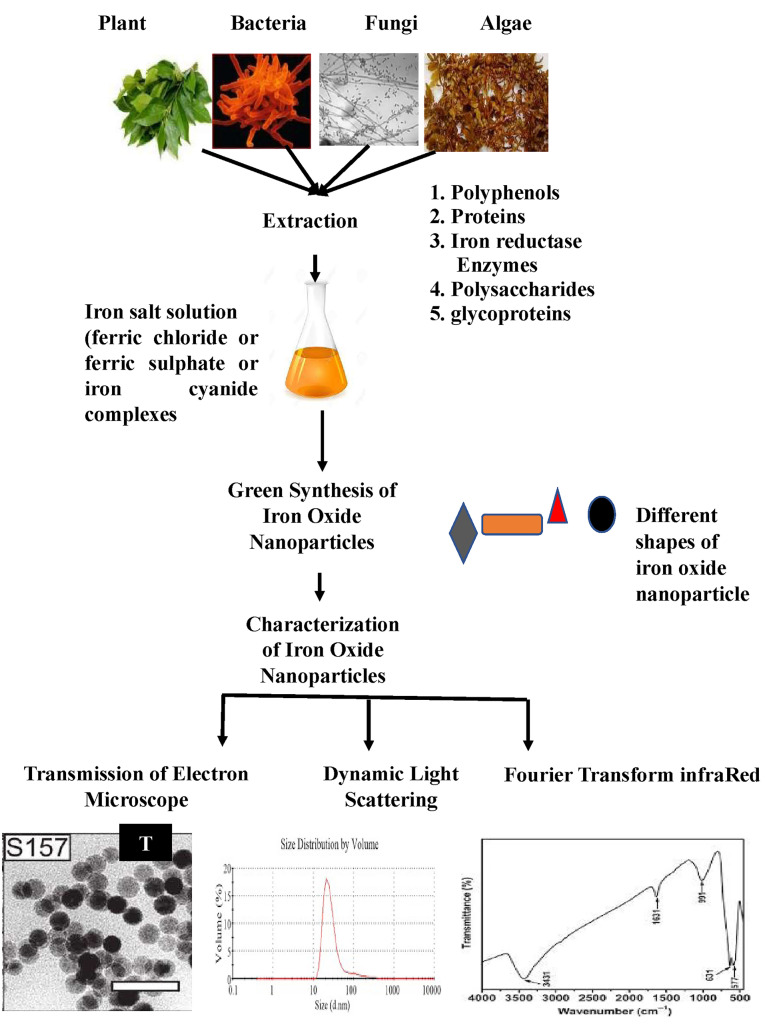
Mechanism of iron oxide nanoparticles biosynthesis.

Biological methods have more advantages over the conventional chemical and physical methods like being non-polluting and eco-friendly. Besides, they have low cost of synthesis since the biological active material acts as reducing and capping agent and produces high yield of small sized nanoparticles (Figure 2). The biological synthesis method aid in iron oxide nanoparticle coating compared to chemical synthesis method.^42^

Many research papers elucidated the biosynthesis of iron oxide nanoparticles (Table 2) with different sizes and shapes from plant extracts such as *Hordeum vulgare* and *Rumex acetosa* extracts. *H. vulgare* contains high amounts of reducing compounds compared to*R. acetosa* extract. However, iron oxide nanoparticles produced by *H. vulgare* were aggregated and unstable. The aggregation and instability problem can be resolved by organic acids in the form of citrate, malate, and oxalate coating. The total protein content and antioxidants properties were similar for the two plant extracts. The stability of iron oxide nanoparticle by*R. acetosa* extract were because of pH 3.7 compared to instability of iron oxide nanoparticle by*H. vulgare* extract which has pH 5.8.^43^

**Table 2:** Biological preparation methods for iron oxide nanoparticles, types of particles, morphology, advantages, and disadvantages of different methods

Biological Methods	**Nanoparticles Morphology**	**Types of** **Particles**	**Advantage**	**Disadvantage**	**References**
Plant - *Hordeum vulgare*	Spherical -30 nm	Mixed iron oxidation states	Eco-friendly	Instability and aggregation of nanoparticles with time	42
Plant - *Rumax acetosa*	Amorphous -40 nm	Mixed iron oxidation states	Eco-friendly Highly Stable	-	42
Plant - *Amaranthus spinosus*	Spherical 91nm	rhombohedral crystalline structure of hematite	Eco-friendly Stable	-	43
Plant - green tea	Spherical 70-80 nm	Maghemite, magnetite and iron hydroxides	Eco-friendly	-	44
Plant - sorghum bran	Amorphous 50 nm	Lack distinct diffraction peaks	Eco-friendly	Agglomeration and irregular clusters	45
Plant - pomegranate	Spherical 10-30 nm	--	No agglomeration	-	46
Brown Algae- *Sargassum muticum*	Spherical-18 ± 4	Cubic form	Eco-friendly-stable -small size	-	49
Green Algae - *Chlorococcum* sp.	Spherical 50 nm	-	Eco-friendly- highly stable	-	50
Fungi - *Aspergillus japonicus*	Cubic 60-70 nm	Magnetite and maghemite	Stable	-	51
Fungi - *Fusarium oxysporum* and *Verticillium* sp	Quasi-spherical 20-50 nm	Magnetite and maghemite	Stable	-	52
Fungi - *Verticillium* sp	Cubo-octahedrally 100-400 nm	Magnetite and maghemite	Stable		52
Bacteria - *Actinobacter* sp.	Spherical 19 nm	Maghemite	Stable	-	53

Also, *Amaranthus spinosus* water leaf extract is added to ferric chloride for the synthesis of spherical iron oxide nanoparticles. The presence of amaranthine and phenolic compounds in this aqueous extract allows the reduction process and capping of iron oxide nanoparticles.^44^ Spherical IONs can be also biosynthesized by using ferric sulphate as precursor and green tea extracts as reducing agent. Characteristic UV peaks are observed at 205 and 272 nm and this is an indication for presence of polyphenols and caffeine in green tea extract. Polyphenols reduce iron salts and is capping it. The diameter of these nanoparticles was 70-80 nm. In general, the reduction potential of polyphenols/caffeine was in 0.3-0.8 V and iron reduction potential was -0.44 V.^45^ Also, adding ferric chloride solution into sorghum bran extract leads to formation of amorphous iron oxide nanoparticles with an average diameter of 50 nm. The polyphenols in sorghum extract stabilizes the biosynthesized iron oxide nanoparticles.^46^

Polyphenols are essential components in the reduction process of iron salts into zerovalent iron oxide nanoparticles because of its antioxidant property.^46^

The possible mechanism for biosynthesis of iron oxide nanoparticles is explained^19^ as follows:

**Figure:**
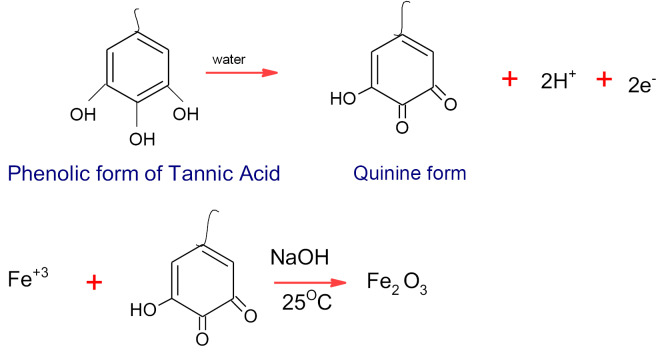


The antifungal features of iron oxide nanoparticles produced by a phenolic form of tannic acid were well studied, which will control fungal diseases.^19^ The use of anhydrous ferric chloride and ferrous chloride hydrate mixtures as a precursor with 6% tangerine peels extract can synthesize spherical iron oxide nanoparticles with an average diameter 50 nm. Increasing the concentration of extract causes sever aggregation of nanoparticles.^47^ Even extracts of several tree leaves such as almond, apricot, avocado, cherry, eucalyptus, kiwi, lemon, mandarin, medlar, mulberry, green tea, black tea oak, olive, orange, passion fruit, peach, pear, pine, pomegranate, plum, quince, raspberry, strawberry, vine, and walnut are investigated for reduction of iron(III) chloride hexahydrate to zero valent iron nanoparticles oxide (d = 10-30 nm). According to their antioxidant activity, green tea, pomegranate and black tea water extracts showed higher antioxidant activity compared to other tree leaves because they are rich with phenolic content.^47^ Moreover, using a polysaccharide template as Chitosan for biosynthesis of spherical -shaped iron oxide nanoparticles is recorded which aided the coating by sand.^42^ Chitosan can change the morphology of iron oxide nanoparticles from rod like, flower like and, cubo-octahedral structures into rice-seed-like, quasi-spherical, and cubic structures, respectively.^49^

Other reports elucidated the mechanism of iron oxide nanoparticles production by sulphated polysaccharide of brown see weeds*Sargassum muticum* extract.^50^ Also soil microalgae *Chlorococcum* sp. can synthesize spherical nano-iron extracellularly and intracellularly. Glycoprotein and polysaccharide mediated the synthesis and stabilization of nanoiron.^51^

On the other hand, fungal protein mediated the biosynthesis of iron oxide nanoparticles. Cationic protein content of *Aspergillus japonicus* isolate AJP01, *Fusarium oxysporum* and *Verticillium sp.* can hydrolyse anionic iron cyanide complexes and produce iron oxide nanoparticles. Nanoparticulate magnetite has size range of 50-60 nm for *A. japonicus* and 20-50 nm for *F. oxysporum* and *Verticillium* sp. ^52,53^ The protein analysis elucidated the presence of two proteins with molecular weight 55 and 13 kDa which are responsible for hydrolysing mixture of iron cyanide complexes and capping of nanoparticulate magnetite.^53^ Also, Iron reductase in bacteria may play role in reduction of iron salt during formation of bacterial maghemite nanoparticles by *Actinobacter* sp. A protein of 55 kD was observed and other new proteins were induced during the biosynthesis process. These new proteins are responsible for capping and stabilization of nanoparticles.^54^

## Properties of iron oxide nanoparticles

There are three types of iron oxide nanoparticles; magnetite, maghemite and hematite. The hematite is red in colour if finely divided or black-grey in colour if crystallized. Magnetite also is black in colour and has strong magnetism. Maghemite is an oxidized metastable product of iron oxide. The instability problem of maghemite at high temperature can be resolved by doping it with other metals. Maghemites can loss its magnetism by irreversible conversion into hematite at around 400°C.^55-58^ Small size of maghemites (<10 nm) is super paramagnetic at ordinary temperature. The magnetic properties of iron oxide nanoparticles are influenced by surface effects. The magnetic properties are lost faster by increasing temperature. Chemical method for surface modification of iron oxide nanoparticles influences their coercivity. The size, nanostructure surface treatments and, method of preparation can change the magnetic properties.^58-62^

Certain sizes, shapes, surface characteristics and magnetic properties of iron oxide nanoparticles are depending upon the used application. The application of iron oxide nanoparticles in biology and medical diagnosis demands the stability of nanoparticles during the physiological conditions.^63,64^ The small dimension of nanoparticles, charge and surface chemistry have influence on stability of colloidal magnetic fluid. Magnetite and maghemite with external magnetic stimuli allow drug delivery and permit low dose administration.^64,65^ Moreover, functionalization of nanoparticles increases therapeutic efficiency.^65^

## Surface modification of magnetic nanoparticles

**Figure 3:**
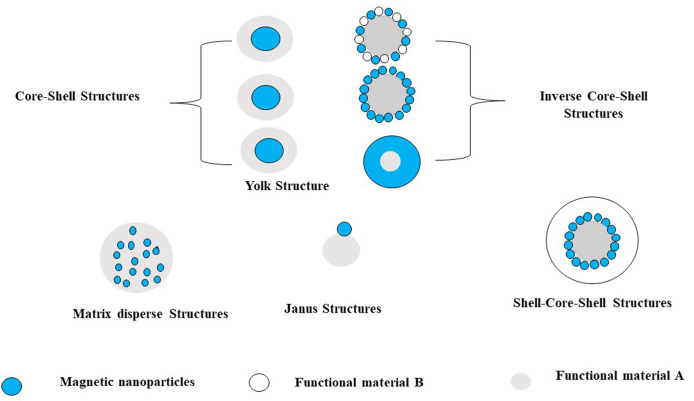
Morphology of magnetic nanocomposites.

Iron oxide nanoparticles may be insoluble and non-biocompatible; Therefore, the surface should manipulate to improve biocompatibility.^66,67^ In general, there are several reasons for surface modification of iron oxide nanoparticles; improvement of the dispersion, surface activity, physicochemical, and mechanical properties can improve the biocompatibility of iron oxide nanoparticles.^67^ There are different shapes of magnetic nanocomposite as in Figure 3.^68,69^

**Figure 4:**
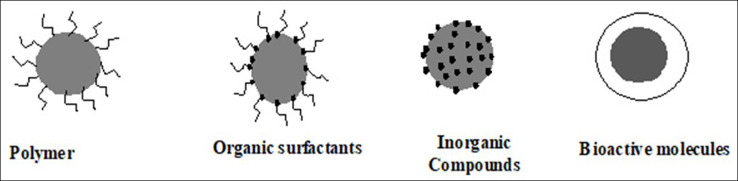
The main shells for manipulating Iron oxide nanoparticle (grey circle).

Several strategies are used for functionalizing iron oxide nanoparticlesfor the stability of colloidal suspension or other desired applications.^70^ Iron oxide nanoparticles can be covered by a shell of organic (surfactants or polymers) or inorganic (carbon or silica) or bioactive molecules as in Figure 4.^23^

*The polymers* can be synthetic as in the forms of polyethylene glycol, polyvinylpyrrolidone, and polyvinyl alcohol or natural as in the form of chitosan.^23,68,71^ The advantage of hydrophilic uncharged polyethylene glycol, when used in the coating of iron oxide nanoparticles, is that it cannot be recognized by the immune system, and this helps to stay in the blood circulation for a long time and gathering in the target organ.^71,72^ In the case of using the hydrophilic polyvinylpyrrolidone, and polyvinyl alcohols which have hydrogel structures so it can be linked with iron oxide nanoparticles by hydrogen bonds, and interactions between polymer and surface can be increased which prevent nanoparticles aggregations.^72^

However, a natural polymer such as chitosan has a positive charge that drives chitosan carriers to negatively charged cell membranes besides their mucoadhesive characteristics, which cause their retention on target cells.^73^ The magnetic and thermal properties of iron oxide nanoparticles cannot be changed by chitosan coating. It was hypothesized that the electrostatic repulsion between the negative potential surface of iron oxide nanoparticles and bacteria lowers the antimicrobial activity compared to the positive potential surface of iron oxide nanoparticles.^74^ However, the partial protonation of amino groups in chitosan coating reduces its water solubility. To overcome such problem, using O-carboxymethyl chitosan or carboxymethyl starch chitosan can be used via some chemical changes to get water solubilization.^23,70,75,76^ Also, sodium alginate as polysaccharide used for grafting magnetic nanospheres and encapsulated by cisplatin to control release the cisplatin dug.^77^

The modification of the shell surface of iron oxide nanoparticles by using a hydrophilic group is one of the most suitable methods for desired applications such as magnetic targeting delivery and hydrothermal cancer therapy.For example,Fe_3_O_4_@ dopamine was used as enzyme mimetic for the detection of bacteria.^78^ Moreover, Iron oxide nanoparticles functionalized with amine groups using (3-aminopropyl) trimethoxysilane. The conjugation of amino with doxorubicin is followed by bonding with bi-functional polyethylene glycol and then folic acid for targeting the tumor. The hydrophobic core is DOX conjugated with iron oxide nanoparticles and polyethylene glycol-OCH_3_/Folic acid, which acts as a shell nanocarriers. Magnetic core aid not only targets the drug for carrying to tumor cells but can also be used for magnetic resonance imaging.^79^

*Non-polymer organic molecules* such as alkanesulphonic or alkanephosphonic acids, oleic, lauric, dodecylyphosphonic, hexadecylphosphonic acids are used for stabilization of iron oxide nanoparticles in organic solvent.^80,81^ However, a long hydrocarbon chain causes the hydrophobic nature of nanoparticles that hinders in vivo applications.^82^

*Inorganic coating materials* like silicon dioxide or carbon are favored in biological labeling or optical bioimaging or in increasing the antioxidant properties. Silicon dioxides coating of nanoparticles maintain the stability of nanoparticles in acidic medium and reduce the toxicity of iron oxide nanoparticles.^83-85^ Also, the carbon coating of iron oxide nanoparticles prevents iron nanoparticles from oxidation besides, the diverse properties of carbon such as stability at different temperatures, good electrical conductivity, and solubility.^71^ The metal coating of nanoparticles prevents the low reactivity of nanoparticles.^68^ Positively charged silver coating allows the conjugation of different antibiotics.^86^ The possible combination between metal oxides creates intrinsic magnetic properties. The selection of coating depends on the purpose of the application. For example, zinc oxide nanoparticle was chosen as a suitable compound for anticancer nano-composite using trisodium citrate as a linker for conjugation of Fe_3_O_4_ with ZnO. The hypothesis for anticancer activity was the reactive oxygen species, which cause the selective cytotoxicity of ZnO and exhaust the activity of scavenging of cancerous cells. Therefore, it promotes the cytotoxicity of iron oxide nanoparticles against cancerous cells.^87^ Moreover, ZnO nanoparticles have the capability of inhibiting pathogenic bacteria, yeast, and filamentous fungi.^88^

*Bioactive molecules* such as lipids, peptides, and proteins can be coated with iron oxide nanoparticles for improving their stability and magnetic properties.^72,76^

## Antibacterial and antifungal iron oxidenanosystems

### Biocidal activity of metals

**Figure 5:**
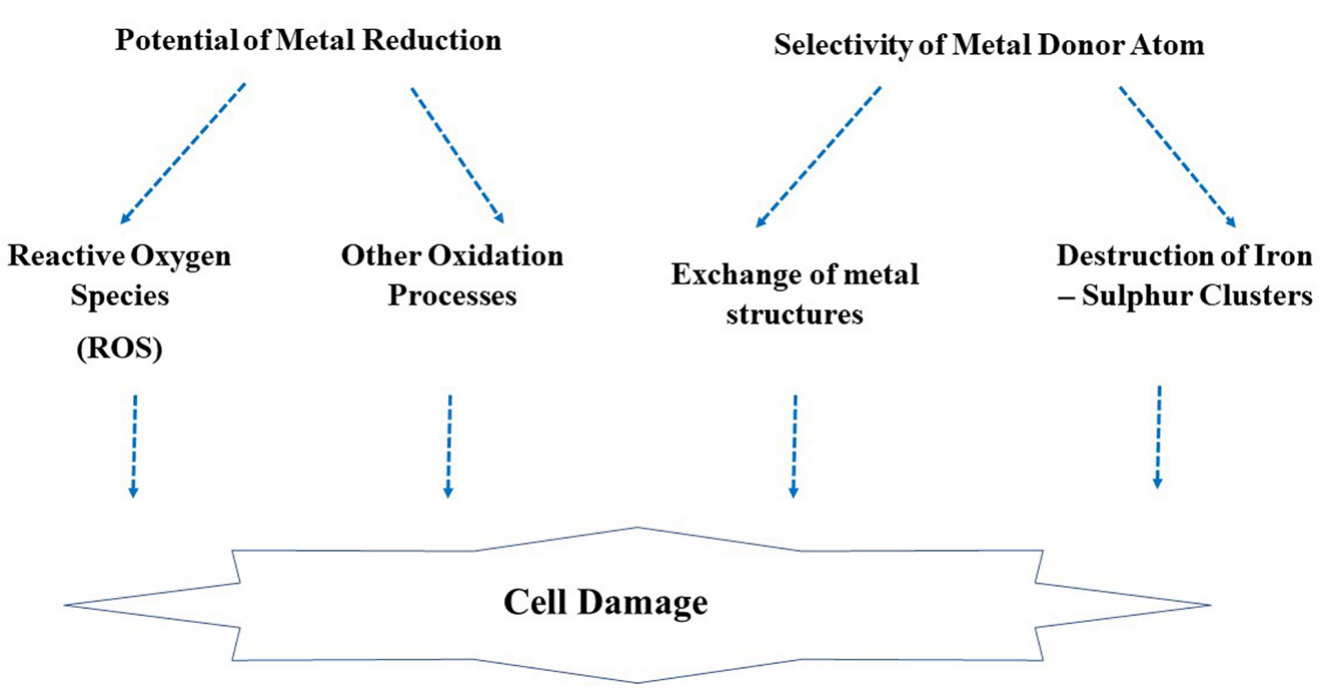
Possible mechanisms behind biocidal activity of metals

Since ancient times, the toxicity of metals is known to bacteria, fungi, and has been used as antimicrobial agents. The possible mechanism is not well elucidated. In general, the biocidal activity of metals depends on the potential of metal reduction and selectivity.^89-91^ The metal toxicity mechanisms (Figure 5) explained as follow:

The potential of metal reduction acts as a cofactor for activating cell enzymes and generation of reactive oxygen species (ROS) that can induce oxidative stress resulting and subsequently in proteins, lipids, and DNA damage. Besides, the excess of ROS induces proinflammatory signals, which cause programmed cell death.^91,92^ The main principle for metal toxicity is the production of reduced forms of oxygen molecules such as hydrogen peroxide and superoxide during aerobic respiration. Hydrogen peroxide can react with metals like iron and produces hydroxide and hydroxide radicals (Fenton reactions). The hydroxide radicals can react with biological molecules such as amino carbon compounds and form carbon-protein radicals or with unsaturated fatty acids and form lipid radicals. Some metals can form protein disulfides by binding with sulfur and causes depletion of glutathione reservoirs. Besides, this mechanism depends upon the selectivity of metal donors, in which the metal ions bind with another atom such as nitrogen, oxygen, and sulfur. Therefore, metal ions or its complexes can replace the original biomolecules metals and causes cell dysfunction. Metals can cause inactivation of enzymes and promote Fe-S clusters.^89,90^ Other mechanisms depend upon cell membranes or intracellular region. For instance, bacterial membranes have highly electronegative macromolecules that are the site for adsorption for metals. Therefore, cell membranes are the first barrier that damaged by metal ions that permit subsequent intracellular uptake and causes bactericidal toxicity.^93^

### Antimicrobial activity of metal nanoparticles

**Figure 6:**
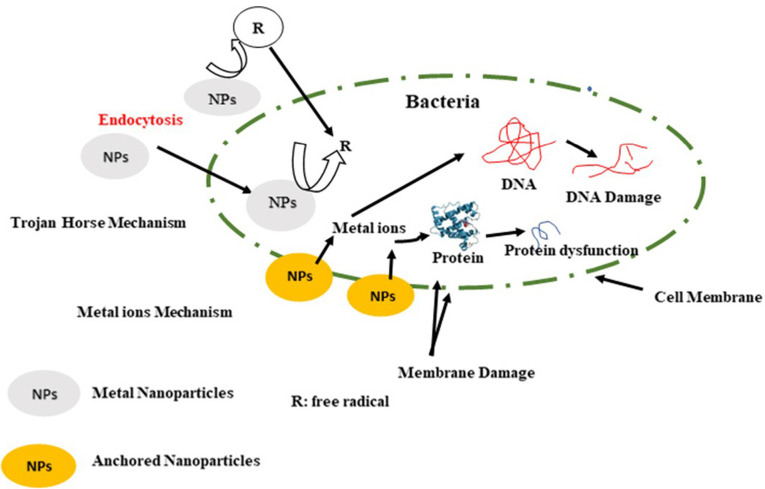
A probable mechanism for antimicrobial effect of metal nanoparticles :1- endocytosis, 2- attachment of membrane surface, 3-free radical formation, and 4- release of metal ions.

Metal nanoparticles should be stronger antimicrobials than metals because of their nanoscale size, and their unique physical and chemical properties. Metal nanoparticles can incorporate directly inside the cell by endocytosis. Hence, the uptake of ions through the cell increases in the form of ionic species and released within the cell. This process is called a Trojan-horse mechanism. Besides the oxidative stress occurs inside the cell.^94^ A probable mechanism for antimicrobial effect of metal nanoparticles is showed in Figure 6.

### Coated and non-coated iron oxide nanoparticles as therapeutic tools to combat pathogenic microorganisms

**Figure 7:**
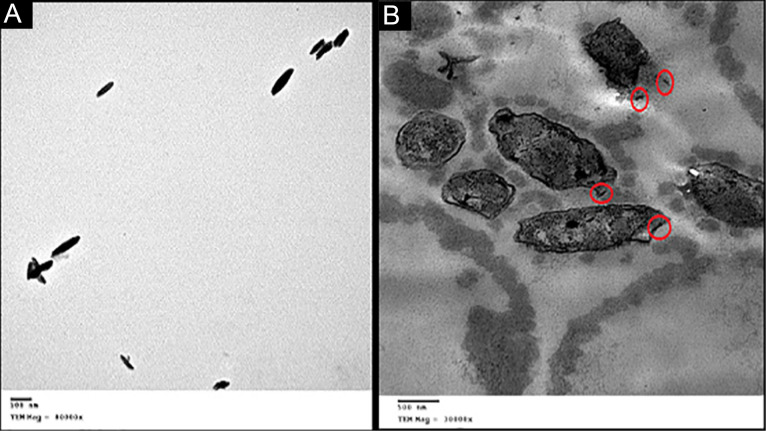
Transmission electron microscope Images of rode shaped iron oxide nanoparticles synthesized by water extract of *Spirulina platensis* (A) and deformation of multidrug resistance *Helicobacter pylori* after treatment with MIC50 of iron oxide nanoparticles (red circle) (B).

Iron oxide nanoparticles adhere to bacterial cell membranes and cause membrane depolarization and loss of membrane integrity. Besides, damage of deoxyribonucleic acid and protein via generation of ROS occurs with lipid peroxidation.^95^ The presence of metal ions inside the cell causes cell imbalance and affects the protein harmony.^96^ Rod-shaped iron oxide nanoparticles synthesized by water extract of *Spirulina platensis* penetrate the cell membrane and cause deformation for the morphology of multidrug-resistant *Helicobacter* pylori (Figure 7).^97^ As a result of continuous leakage of intracellular content and shrinkage of the cell membrane, the death of bacteria occurs.

Carboxylate functionalized iron oxide nanoparticles penetrate the biofilm of bacteria and reduce their growth.^98^ Also, gold-coated iron oxide nanoparticles can adhere to the bacterial protein by disulfide bonds and influence the on bacteria metabolism by increasing the permeability of cell membranes causing damage to the bacterial cell wall. Changes in the morphology of *Pseudomonas aeruginosa* can occur due to the interaction of gold-coated iron oxide nanoparticles with protein F, which has the main role in the resistance of bacteria against antibiotics.^99^ Magnetic iron oxide nanoparticles can catch gram-positive and gram-negative bacteria because of the presence of protein F in both.^100^

Metals can be incorporate on polymer surface or impregnated into the matrix. These materials possess both antibacterial and antifungal activities. The antimicrobial mechanism of polymer@ metal nanocomposite depends on metal nanoparticles and free metal ion received from metal nanoparticles. Several reports recorded the importance of released metal ions in the antimicrobial activity of polymer@ metals nanocomposite.^101^ Microorganisms can form a biofilm to adhere to the biomaterial surfaces and protect itself from antibiotics and host defence mechanisms. The biofilm growth can be reduced in the presence of a polymer brush combined with a high concentration of iron oxide nanoparticles.^102^

Combination with metal nanoparticles is considered as an alternative approach to overcome the resistance of microorganisms to the antibiotics.^103^ Therefore, loading nystatin antifungal drugs on chitosan-coated iron oxide nanoparticles showed a comparable enhancement in fungal activity against *C. albicans* . Besides, it showed better antimicrobial activity against *P. aeruginosa* and *Escherichia coli* than *Staphylococcus aureus* .^104^ Also, the conjugation of two polyene antibiotics such as amphotericin B and nystatin to magnetic nanoparticles increase the antifungal/antibiofilm activity against clinical isolates of *Candida* species. The mechanism of antifungal/antibiofilm activity has been investigated as the cause for inactivation of catalase and imbalance of oxidation-reduction that inhibits *Candida* growth. Hemolytic activity of polyene antibiotics against human red blood cells decreased after magnetic nanoparticle conjugation.^105^ A group of researchers prepared two magnetic nanocomposites @ silver nanoparticles by using a polyacrylate linker. Nanocomposites possess significant antibacterial and antifungal activity against different bacteria strains and *Candida* species.^105^ In that concern, Prucek et al thermally synthesized iron oxide nanoparticles conjugated with silver nanoparticles with good antimicrobial activities that can be used in biomedical applications as disinfectants.^106^ Also, Wilczewska et al investigated that the conjugation of magnetic nanocarriers with metallocarbonyl complexes showed good antifungal activity against *C. albicans* .^107^

## Conclusion and Future Prospects

The Surface coating of Iron oxide nanoparticles not only decreases the cytotoxicity of iron oxide nanoparticles but also increases the stability and efficiency of antifungal and anticancer properties of nanoparticles. The coating of Iron oxide nanoparticles with metal or other metal oxide nanoparticles may even cause a revolution in the therapeutic world.

## Ethical Issues

Not applicable.

## Conflict of Interest

Authors declare no conflict of interest in this study.

## Acknowledgments

Authors thankfully acknowledge the financial support provided by FICCI, DST, New Delhi, Government of India [DCS/2018/000048], Asian Research Training Fellowship Scheme for Developing Country Scientist (RTF-DCS), and Bharathidasan Institute of Technology, Anna University, Tiruchirappalli-620024.Tamilnadu, India, and the Microbiology Department, National Organization for Drug Control and Research, Giza, Egypt.
